# A Novel Performance Assessment Approach Using Photogrammetric Techniques for Landslide Susceptibility Mapping with Logistic Regression, ANN and Random Forest

**DOI:** 10.3390/s19183940

**Published:** 2019-09-12

**Authors:** Eray Sevgen, Sultan Kocaman, Hakan A. Nefeslioglu, Candan Gokceoglu

**Affiliations:** 1 Department of Geomatics Engineering, Hacettepe University, 06800 Beytepe Ankara, Turkey; eraysevgen@hacettepe.edu.tr (E.S.); sultankocaman@hacettepe.edu.tr (S.K.); 2 Department of Geological Engineering, Hacettepe University, 06800 Beytepe Ankara, Turkey; hanefeslioglu@hacettepe.edu.tr

**Keywords:** landslide susceptibility performance, random forest, artificial neural network, logistic regression, photogrammetry

## Abstract

Prediction of possible landslide areas is the first stage of landslide hazard mitigation efforts and is also crucial for suitable site selection. Several statistical and machine learning methodologies have been applied for the production of landslide susceptibility maps. However, the performance assessment of such methods have conventionally been carried out by utilizing existing landslide inventories. The purpose of this study is to investigate the performances of landslide susceptibility maps produced with three different machine learning algorithms, i.e., random forest, artificial neural network, and logistic regression, in a recently constructed and activated dam reservoir and assess the external quality of each map by using pre- and post-event photogrammetric datasets. The methodology introduced here was applied using digital surface models generated from aerial photogrammetric flight data acquired before and after the dam construction. Aerial photogrammetric images acquired in 2012 and 2018 (after the dam was filled) were used to produce digital terrain models and orthophotos. The 2012 dataset was used for producing the landslide susceptibility maps and the results were evaluated by comparing the Euclidian distances between the two surface models. The results show that the random forest method outperforms the other two for predicting the future landslides.

## 1. Introduction

Although the Earth’s surface is dynamic and landslides are part of the dynamic processes, they are sometimes triggered by human intervention, especially for infrastructure construction purposes. Constructing a dam is an important interference to the topography and other nature characteristics of a region. In addition, proper site selection for such an expensive investment is crucial for its construction costs and life time. Landslides can be triggered by dams, and in return, they may affect the life time of dams. Therefore, producing accurate landslide inventories and reliable susceptibility maps are needed prior to site selection.

During the last three decades, thousands of landslide susceptibility maps have been produced and published in international literature. Several auto-production methods [1,2,3] for landslide susceptibility maps have been applied in the literature and their performances have generally been compared by employing landslide inventories that are prepared manually. However, the main purpose of a landslide susceptibility map is to assess the probability of landslide occurrence in an area and depict the findings spatially. In other words, a landslide susceptibility map aims at predicting any future landslide activity. For this reason, the performance assessment of a susceptibility computation method using existing landslide inventory is a little bit open to discussion. However, it has been almost impossible to assess the performance of a landslide susceptibility map up to now due to the lack of historical information and records. Additionally, according to [4], one of the emerging priorities and new frontiers in engineering geology is validation of models in order to provide more quantitative and reliable results. Gokceoglu and Sezer [5] stated that the increasing trend in the regional landslide susceptibility and hazard assessments is expected because regional landslide susceptibility assessment is considered as the first stage of landslide hazard mitigation efforts. A recent review on landslide susceptibility literature [6] has shown the rising trend in the number of publications on this topic. Despite the increasing number of studies, validation of the methods and the susceptibility maps rely only on the existing landslide inventories.

Among several landslide susceptibility map production methods proposed in the literature, artificial neural network (ANN) ([7,8,9,10,11,12]) and logistic regression (LR) (i.e., [13,14,15,16,17,18,19,20]) methods were widely used and often found successful. On the other hand, the application of random forest (RF) method is relatively new and rare (i.e., [21,22,23,24,25,26]). In this study, these three data-driven machine learning (ML) methods were selected for landslide susceptibility mapping and performance comparison. The main goal of this study is to assess the performances of the landslide susceptibility results obtained from these methods (i.e., ANN, LR, RF) in close vicinity of a recently built dam reservoir area in Turkey. Using the 2012 photogrammetric data, landslide susceptibility models were produced. Model training and prediction performances were evaluated separately. The model training performances of three different machine learning algorithms were controlled by the Receiver Operating Characteristic (ROC) curve approach. On the other hand, the prediction performances of the models were evaluated by a novel process. First, the comparison of the surface models of 2012 and 2018 was established. The Euclidean distance approach was used for this purpose. Depending on the Euclidean distance evaluations, surface deformations were determined. New landslides and new activities within the existing failures were defined by investigating the surface deformations observed between 2012 and 2018. As a result of this analysis, the distribution of the post-2012 landslide activities in the landslide susceptibility classes of the 2012 models were determined. This distribution corresponds to the prediction performance of the landslides susceptibility models of 2012. In the models having higher prediction performances, it is expected that the post-2012 landslide activities are observed in areas having higher landslide susceptibility values. As is widely known, slope instabilities around dam reservoirs can be triggered by water, and expected landslides serve to achieve the aims of this study by eliminating the need for natural triggers. The study area covers Kalekoy Dam, which is located in Bingol Province in the Eastern part of Turkey. The reservoir area is located in an active tectonic area and has steep and young morphology in a mountainous region. Consequently, the case investigated in the present study is one of the rare examples for performance assessment of regional landslide susceptibility approaches.

## 2. Characteristics of the Study Area

The study area, Kalekoy Dam, is located in an active tectonic valley and is limited by the lithological boundary of Late Miocene-Pliocene aged undifferentiated volcanic rocks [27]. Volcanic rocks are mainly composed of basalt, andesite, and pyroclastic layers [28]. It sometimes includes sedimentary intercalations, and is partly built from volcanic sediments [28]. The major geological structure observed in the study area is the Kalekoy segment of the Kavakbasi right lateral strike slip active fault [29]. As mentioned, the study area is an active tectonic valley mainly controlled by the Kalekoy segment. According to the Köppen climate classification, a “Dsa” climate is observed in the study area. “Dsa” means the winters are cold and the summers are arid hot [30]. The mean annual precipitation is given as 701 mm [31]. Due to high seismicity and active tectonism, the topography is very young and steep, and the lithological units have weak shear strength characteristics. Combination of these two adverse factors makes the region prone to landsliding. Solhan volcanic rock units constitute the most landslide-prone lithologies in the regions. The transformation of tuffs into clay by weathering processes cause a considerable reduction in shear strength. Additionally, due to the degradation and weathering processes of basalts, clays are also formed, and these clay layers constitute impermeable layers in the formation. Therefore, percolated surface water and groundwater are able to be accumulated on these layers and increase pore water pressures in the weathered parts of the Solhan volcanic rocks in particular. This simplified process causes many landslides in the region.

**Figure 1:**
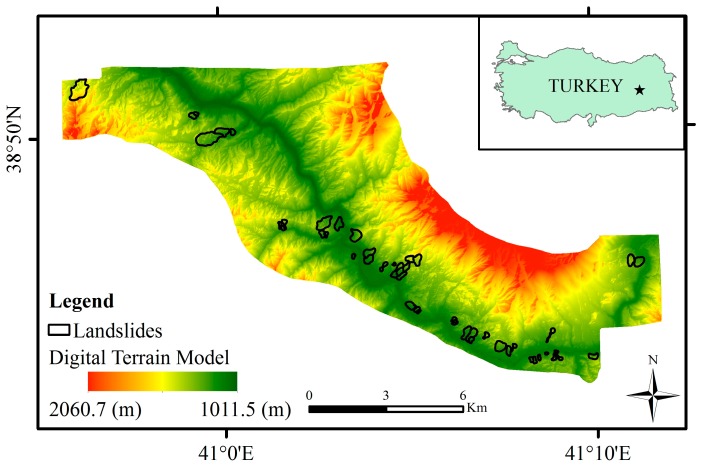
Color-coded digital terrain model (DTM) (from year 2012) of the study area and the landslides.

Figure 1 depicts location of the study area, the color-coded digital terrain model (DTM) of the year 2012 (before the dam construction), and the landslide inventory. The landslide inventory has been prepared manually using the orthophotos and the DTM of the year 2012. The area has been delineated according to a single lithology type to ensure that the whole area has the same lithological characteristics.

**Figure 2:**
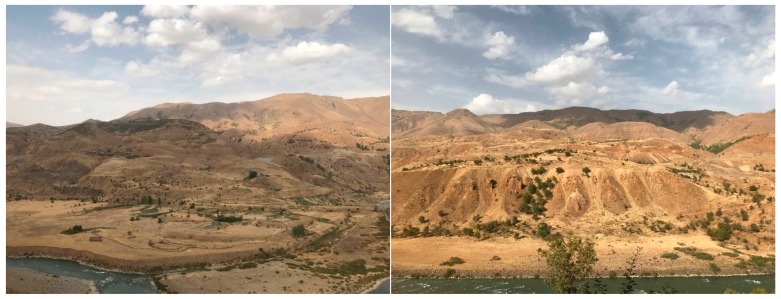
Photos showing the typical landslide morphology in the study area (Photos from the area taken before the dam construction).

A total of 46 landslides occurring in the Solhan volcanic rocks were detected in the study area as shown in Figure 1. Although the landslide inventory of the study area was prepared from the DTM and the orthophotos, an extensive field study was also carried out in order to ground control of the landslide inventory (Figure 2). The landslides were classified as slide according to the classification system suggested by [32]. Additionally, according to [32], the activity states are mainly defined as dormant, the distribution of the activity is defined as retrogressing, and the main activity style is defined as successive. The minimum landslide area was calculated as 9725 m^2^, while the maximum landslide area was found to be 382,327 m^2^ (Table 1). To determine the landslide volumes, the empirical equation suggested by Larsen et al. [33] was implemented. Accordingly, the minimum and maximum landslide volumes were determined as 29,941 m^3^ and 802,017.7 m^3^, respectively. Considering the landslide dimensions defined by IAEG [34], the expected depths of failures were also determined. The minimum depth value was calculated as 19 m while that of maximum value was found to be 63 m. The mean depth value of all failures observed in the study area was calculated as 33 m. An additional landslide mapping was also carried out using the DTM produced from ortophotos taken in 2018. Both new occurrences of landslides and activities on the existing failures were determined in the data of 2018. The comparisons performed of which details are given in further sections of this paper, justify the landslide activity definitions given up to here.

## 3. Methodology and Data

**Figure 3:**
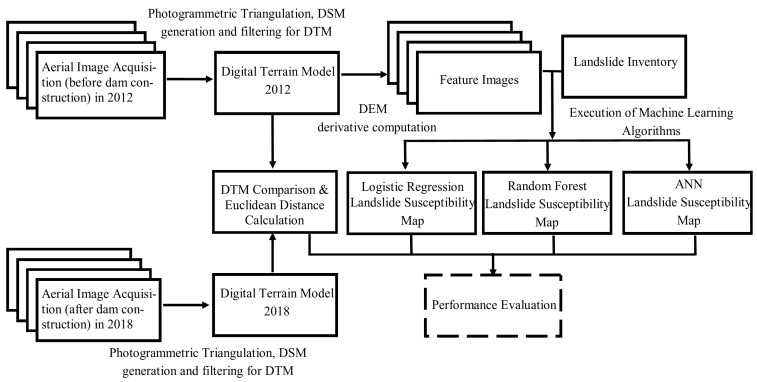
Overall workflow of the methodology.

A generic workflow of the study is provided in Figure 3. Aerial photogrammetric images acquired in two different years, 2012 and 2018, using a large-format aerial camera, were obtained from the General Directorate of Mapping (GDM) in Turkey together with image orientation and camera calibration parameters. The datasets were processed with photogrammetric methods to generate dense point cloud, high resolution digital surface models (DSMs), and orthophotos. The DTMs were produced from the DSMs through point cloud filtering. The buildings and the vegetation were removed in a filtering process and the remaining surface models include the terrain surface only. The topographic (or digital elevation model, DEM) derivatives, such as slope, aspect, curvature, etc., were computed from the DTMs. The landslide inventory and the feature images obtained from the topographic derivatives were used to produce the landslide susceptibility maps of the area by using the RF, LR, and ANN techniques. The DTMs of 2012 and 2018 were compared using the least squares surface matching and comparison approach developed by [35]. The results of the DTM comparison, which reflect the landsliding caused by the dam, were used for the visual inspection of the performances of the ML methods. A quantitative assessment is not yet possible since some of the landslides have been filled with water. More details on the data and the methods are given in the following sub-sections.

### 3.1. Photogrammetric Processing

Aerial photogrammetric flight missions have been regularly performed by GDM in Turkey since 1939 [36]. Although the standard outputs involve manually measured DTM and orthoimages, raw (geometrically and radiometrically unprocessed) images and their interior orientation (IO) and exterior orientation (EO) parameters were obtained from the GDM in order to produce dense DSM and DTM of the area. The IO (camera calibration) parameters provided by the camera vendor (Ultracam Eagle from Vexcel Imaging, Graz, Austria) and the EO parameters measured by onboard GNSS and INS instruments have been refined in a bundle block adjustment process using sufficient number of ground control points for every photogrammetric project. Therefore, the adjusted IO and EO parameters delivered by GDM ensure photogrammetric point positioning accuracy of better than one pixel (i.e., sub-pixel accuracy) as per the national map production standards using aerial photogrammetric methods in Turkey.

Data from two different flight missions, i.e., performed in years 2012 and 2018, were employed in this study. The main flight parameters are provided in Table 2. The ground sampling distances (GSD) of the photos vary due to the altitude variances of the terrain (ca. 1000–2100 m). The triangulation accuracies obtained from these photogrammetric blocks are around 1/3 pixel in planimetry and 1/2 pixel in height (ref.: personal communications with GDM personnel). The images and the orientation parameters were used for the generation of dense point cloud and orthoimage mosaics. The output DSMs were filtered to obtain DTMs, and then down-sampled by spatial interpolation to 10 m grid DTMs in order to achieve computational efficiency in the latter steps.

### 3.2. Topographic Derivatives

In the present study, only topographic parameters (i.e., derivatives computed from DTM of 2012) were employed. The area within the unique lithology was evaluated. Almost all of the landslides observed in the field are observed within this unit. As mentioned, the Solhan volcanics are prone to landslides in terms of their rock weathering and structural properties. The approach is that, when lithology is accepted as unique, the landslide susceptibility is controlled by the topographic parameters that vary depending on rock weathering and structural properties. In other words, the topography represents the rock mass strength characteristics that vary depending on rock weathering and structural properties. The Solhan volcanics are located in the Bingol complex and the East Anatolian Fault Zone. For this reason, these parameters were not considered when performing landslide susceptibility assessments. Among the topographic derivatives, slope, aspect, altitude, stream power index, distance to channel, distance to ridge, stream power index, general, plan, and profile curvatures were considered as conditioning parameters during the landslide susceptibility analyses.

**Figure 4:**
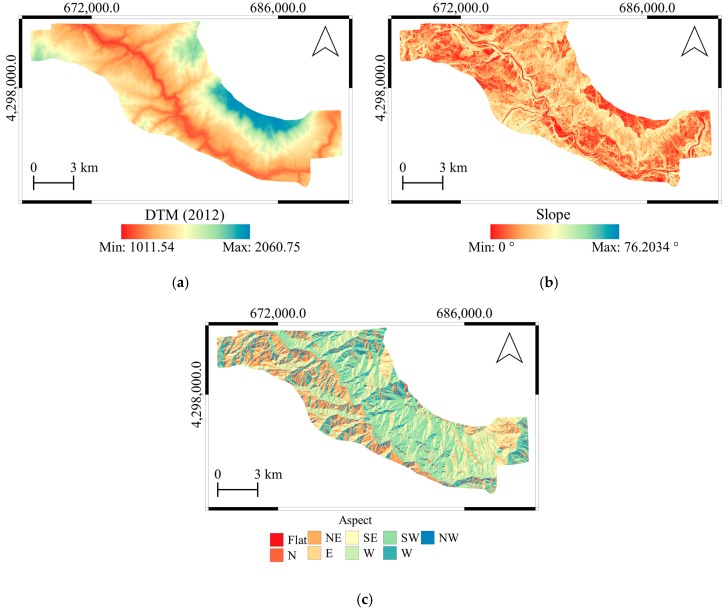
Spatial distribution of (**a**) altitude in meters, (**b**) slope in degrees, and (**c**) aspect as geographical directions. The parameters have been calculated from the DTM of year 2012.

Figure 4 shows the spatial distributions of the altitude, slope and aspect calculations from 2012 DTM. The main stream in the study area is Murat River and the general physiographic trend of the study area NW-SE. The lowest altitude is 1012 m while the maximum is 2061 m. Especially the northeast parts of the area show the maximum altitudes. The highest slopes are observed around Murat River and the north eastern parts of the area. The reservoir slopes are relatively high and therefore more susceptible to landsliding. Depending on the general physiographic trend of the area, the major slope aspects have SW and NE directions.

The parameter the stream power index (SPI) simply defines erosive power of flowing water based on the assumption that discharge is proportional to specific catchment area [37]. In order to evaluate the SPI parameter, Moore et al. [38] propose the following equation (Equation (1)):

(1) $$SPI=A_{s}\times tan\beta$$

where A_s_ is the specific catchment area (m^2^ m^−1^), β is the slope gradient (°).

**Figure 5:**
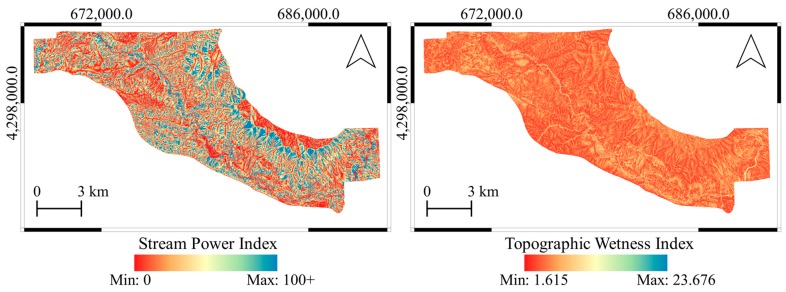
Stream power index (SPI) and topographic wetness index (TWI) maps of the study area produced from 2012 DTM.

Topographic wetness index (TWI) defines the effect of topography on the location and size of saturated source areas of runoff generation [38]. The parameter TWI has been significantly investigated in landslide susceptibility mapping and constitutes one of the most common used conditioning factors [39]. Moore et al. [38] also suggested an equation for the calculation of TWI under assumed steady-state conditions and uniform soil properties (Equation (2)). Both the SPI and TWI maps of the study area provided in Figure 5.

(2) $$TWI=ln\left(\frac{A_{s}}{tan\beta }\right)$$

**Figure 6:**
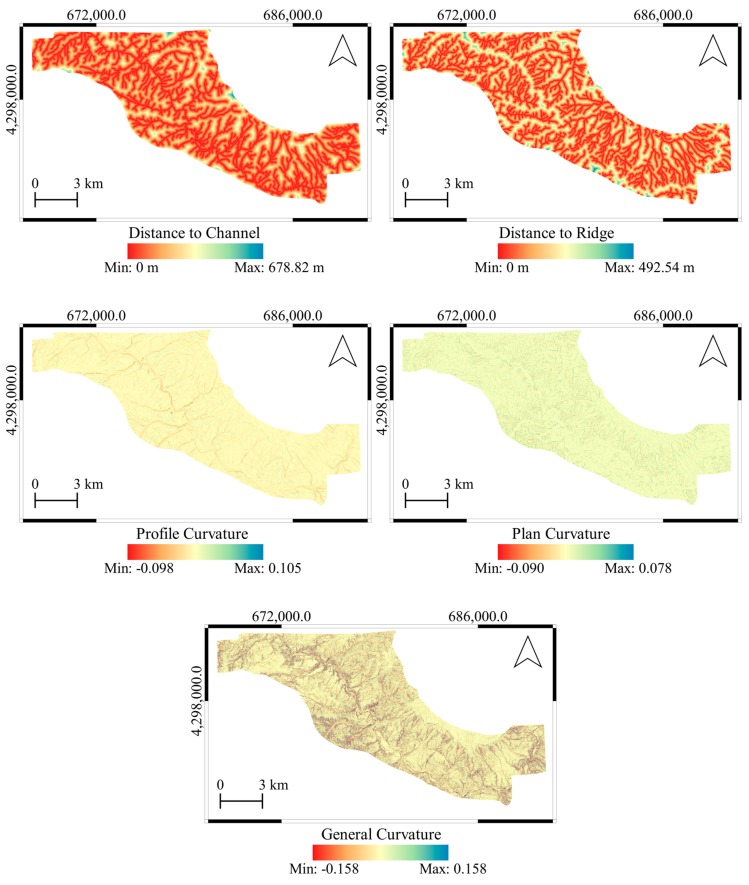
Distance maps to the channels and the ridges and the curvature (general, plan, and profile) parameters produced from 2012 DTM.

**Figure 7:**
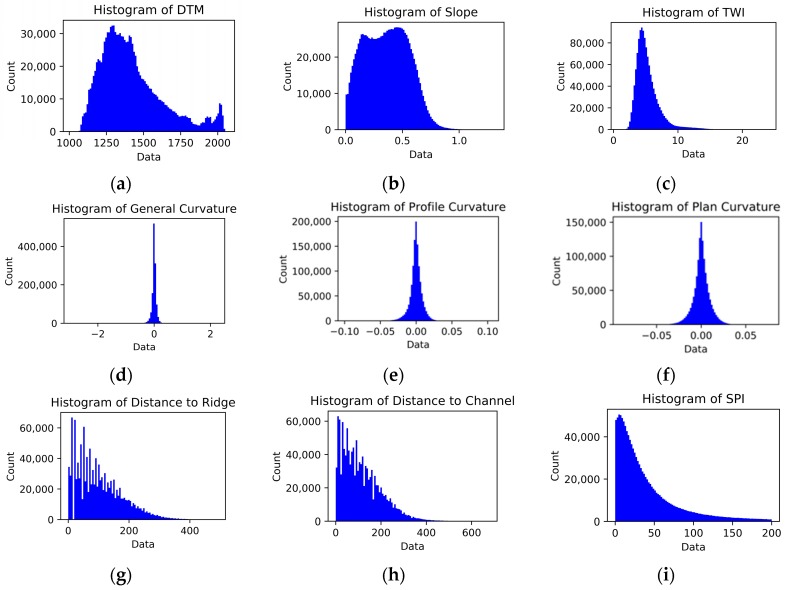
Histograms of topographical parameters derived from 2012 DTM.

Slope curvatures are the second derivatives of DTMs. The curvature parameters define the rate of change of the first derivatives slope gradient and slope aspect in a certain direction [37]. In addition, the distances to the channels (drainage) and the ridges are also effective parameters on the landslide susceptibility and have been employed in previous studies [7]. The curvature maps (general, plan, and profile) and distance maps to the channels and the ridges produced from 2012 DTM are shown in Figure 6. The overall statistical values calculated from the topographic derivative parameters are given in Table 3. In addition, the histograms of all derivative calculations are shown in Figure 7.

### 3.3. Application of the ML Algorithms

LR, ANN, and RF are widely used supervised learning algorithms in machine learning. Supervised learning methods in principle use data samples and estimate the model parameters based on those data samples. Particularly for landslide susceptibility mapping, the model learns from actual landslide areas and no-landslide areas and gives predictions for the future landslides, known as probability of occurrence of landslide. On the other hand, one can consider landslide susceptibility mapping as a binary classification in supervised learning where each input pixel is actually labeled as 0 and 1. This classification output refers the landslide occurrence probability of each pixel in the whole dataset.

The method of LR relies on well-known sigmoid function and gives the probability of landslide occurrence in the range of 0 and 1. Many references can be found on the details of LR method [40,41]. The formula for logistic regression is given in Equation (3).

(3) $$P(y=1|x;\theta )=\;\frac{1}{1+e^{-\;\theta x^{T}}}$$

where θ shows parameter vector, $\left[\theta_{0},\;\theta_{1},\;\theta_{2},\;\ldots ,\theta_{n}\right]\;$, *x* shows feature vector, $\left[1,\;x_{1},\;x_{2},\;\ldots ,x_{n}\right]{\;}^{T}$.

Using independent variables x, parameter vector θ is estimated iteratively with respect to a cost function. In this study, the cost function is binary cross entropy with L2 regularization as shown in Equation (4). Parameters estimated based on this cost function and updated in each iteration.

(4) $$J\left(\theta \right)=\;\overset{n}{\underset{i=1}{\sum }}-(ylog\left(p\right)+\left(1-y\right)log\left(1-p\right)+\;\frac{\lambda }{2}\;\overset{m}{\underset{j=1}{\sum }}\theta^{2}$$

where p refers likelihood of the occurance of the landslide, y shows desired value, and λ is the regularization strength. 

**Figure 8:**
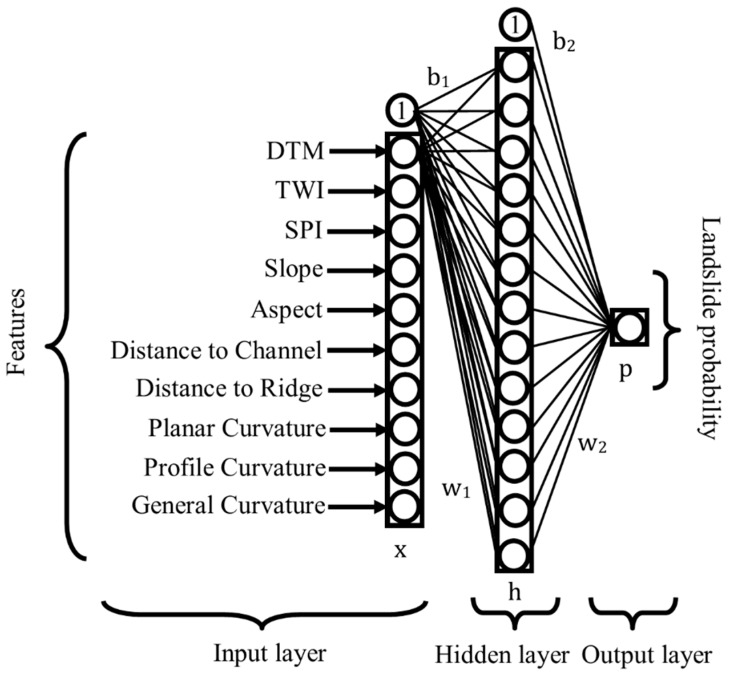
The ANN algorithm used in the study.

ANN is a more complex model in comparison to LR and aim at iterative determination of the weights with respect to the given true data samples. In the training stage, the network predicts each sample’s label using their weights and bias values. A cost function finds the distinction between the calculated label and true label. At the backpropagation stage, each weight receives an update according to gradient of the cost function. This process continues until a convergence is met or a maximum number of epoch is reached in the training. More details on the ANN method can be found in [42]. In Figure 8, a visually simplified version of ANN architecture used in this study is given.

According to Figure 8, the input layer has totally 10 neurons. In other words, the parameters are optimally estimated in a 10 dimensional space. The network has one hidden layer with 20 neurons. The last layer has only one neuron, which shows the landslide occurrence probability. The mathematical relationship is denoted in Equation (5).

(5) $$\begin{array}{c} h\;=f\left(\;x\;w_{1}+\;b_{1}\right)\\ p=hw_{2}+\;b_{2} \end{array}$$

where

- x refers feature matrix, the size of the matrix is k by n, where n is feature size, k is sample size;
- w refers weights, the size of the weight matrix depends on the number of features and number of neurons in the hidden layer (or the number of neuron in the next layer);
- b refers bias vector, the size of the vector dependent on the number of neurons in the next layer;
- p refers output classification score between zero and one;
- f() is sigmoid activation function.

A few factors influence the success of the neural network applications. The first one is the dimensionality of the data. With a higher number of dimensions, more complexity occurs in the network although it helps to solve more complex classification problems. The second factor is the numbers of hidden layers and the number of neurons in the hidden layers. According to the heuristic approaches, doubling the number of features is a good choice for neural networks with one hidden layer. The third and the most important factor is the normalization of the data. In other words, all data should be in the same scale. Although neural networks are able to find a solution with non-normalized data, it takes longer and is not guaranteed to reach the global minimum of the system.

Random forests [43] can be shortly defined as an ensemble method of decision trees. It creates decision trees selecting randomly from the training data set. At the training stage, the decision trees have been evaluated separately and the best score is the average of the results of the trees. The feature selection plays a key role because RF tries to select the most important features when creating decision trees. There are a few important parameters for RF. The first one is the number of trees in the forest, which defines how many decision trees are created in the training. The general approach to set this parameter is that a large number of trees help to generalize the model better. Another parameter is the number of features, which refers the depth of the decision trees. With a larger number of features, better accuracy can be obtained. It should be noted that larger values increase the computational time and one should therefore consider the time in parameterization.

All three ML models were implemented in Python programming language using Scikit-learn [44] ML library. The parameters for each model are summarized in Table 4.

### 3.4. Performance Assessment Approaches to the Landslide Susceptibility Maps

Two main performance assessment approaches were implemented in this study. The first one is the conventional internal accuracy assessment approach based on the visual analysis of the Receiver Operating Characteristic (ROC) curves and the area under curve (AUC). The ROC curve has been a common way of visualizing a classifier’s performance, and the AUC is one of the best single number indicator [45]. According to the AUC, a larger area shows the better classification accuracy.

As the second approach, the performance of the landslide susceptibility maps was assessed externally by analyzing the landslides occurred after the susceptibility maps were produced. For this purpose, the susceptibility maps produced using the 2012 dataset were assessed with the terrain deformation information derived using 2012 and 2018 datasets. A least square surface matching algorithm and tool, i.e., LS3D, developed by [35] and later used for diverse applications ranging from cultural heritage and object modelling to geomorphology [46,47,48,49,50,51], was used here. Since 1D height differences along the *z*-axes may not truly represent the surface-to-surface distance, the Euclidean (3D) distances as provided in LS3D were analyzed here. Although the algorithm is powerful enough to estimate the transformation parameters between two or more 3D surfaces by minimizing the sum of the squares of the Euclidean distances between the surfaces, no surface matching was performed in this study since the absolute orientation of the photogrammetric datasets were very accurate. 

## 4. Results 

**Figure 9:**
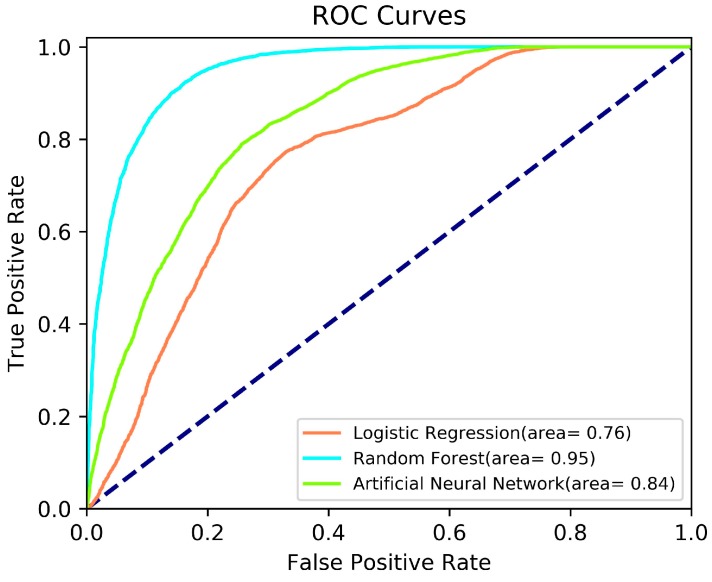
ROC curves obtained from the classifications with LR, ANN, and RF.

The ROC curves [52] and the AUCs for each model are demonstrated in Figure 9. This assessment method was used previously by several authors [53,54,55,56] to check the model performance. In the present study, the ROC curves were also used for same purpose. As shown in Figure 9, the RF method yields the best classification accuracy with an area value of 0.95, where ANN comes second in the performance with an area of 0.84. The LR algorithm leads to the least accurate results. Assessment of the ROC curve (Figure 9) shows that all ML approaches employed in the present study provide acceptable results.

The landslide susceptibility maps and the surface comparison results between 2012 and 2018 DTMs were provided in the following sub-sections for further visual analysis. As a novel performance assessment, the resulting maps and orthophotos in some parts of the areas were draped on the DTMs in a 3D geovisualization software.

### 4.1. Landslide Susceptibility Maps

**Figure 10:**
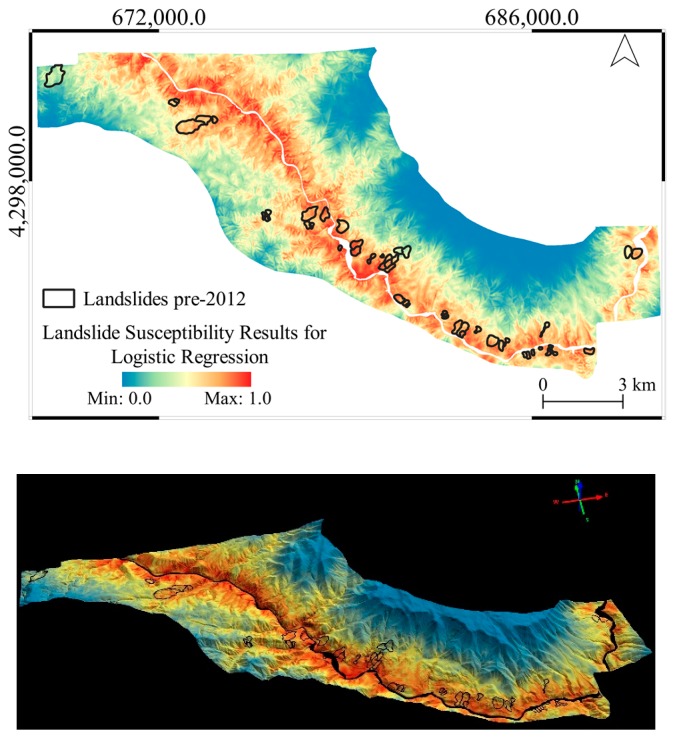
The landslide susceptibility map produced with the LR method in 2D (above) and 3D (below) visualization.

**Figure 11:**
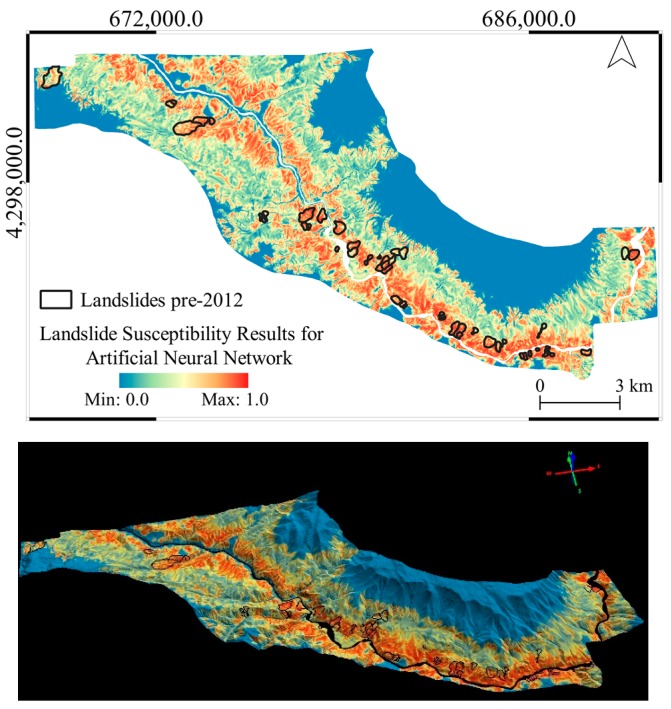
The landslide susceptibility map produced with the ANN method in 2D (above) and 3D (below) visualization.

**Figure 12:**
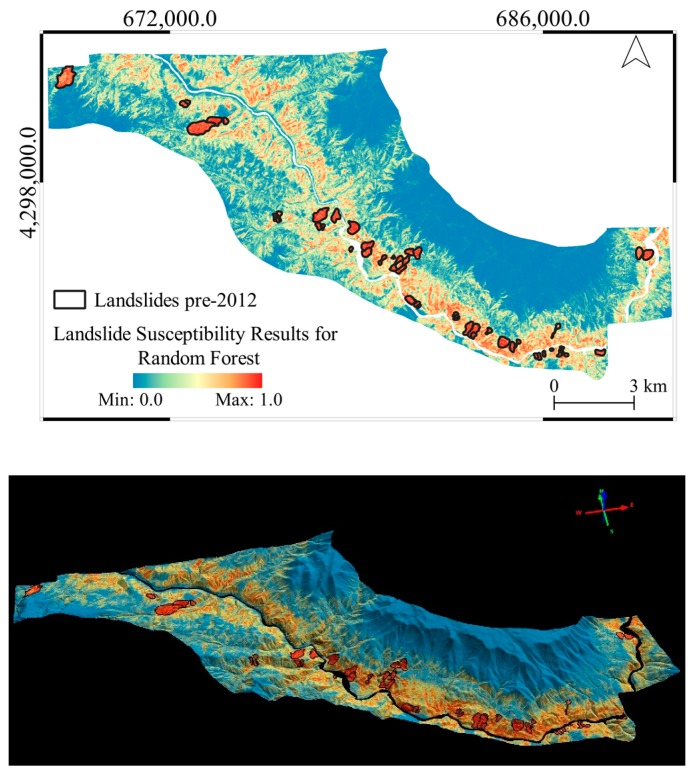
The landslide susceptibility map produced with the RF method in 2D (above) and 3D (below) visualization.

The general overviews of the produced landslide susceptibility maps are provided in Figure 10, Figure 11 and Figure 12 in both 2D and 3D. From the figures, it can be seen that LR produces a very similar output to the altitude classification map (DTM) from 2012, as shown previously in Figure 4.

### 4.2. Surface Comparison Results

**Figure 13:**
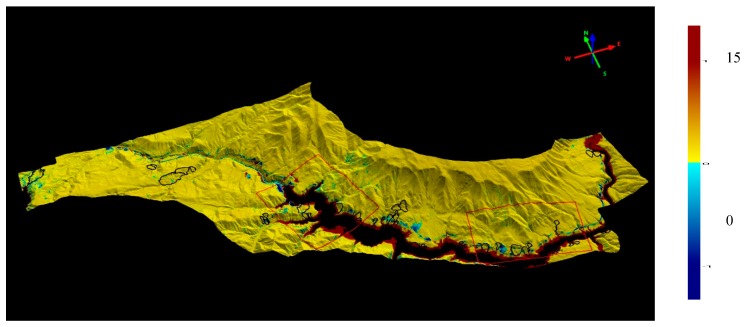
The residual plot of the Euclidian differences obtained from the comparison between 2012 and 2018 DTMs of the study area.

**Figure 14:**
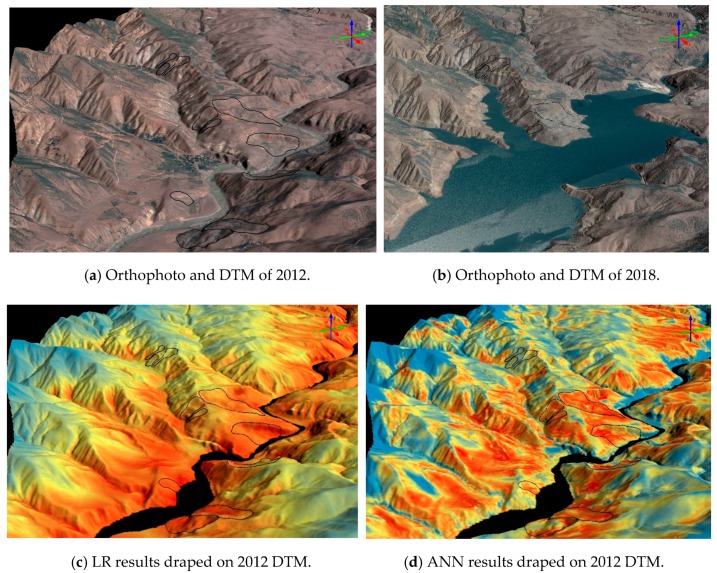

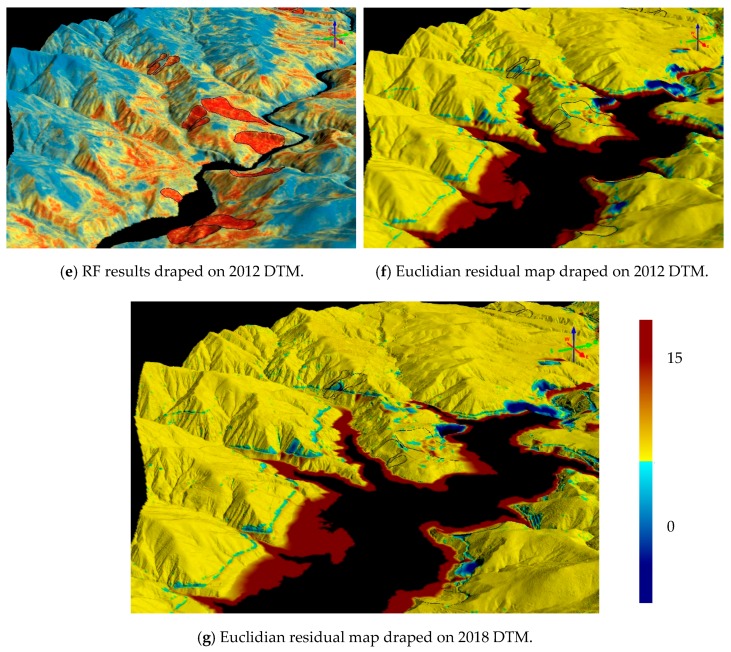
The ortophotos (**a**,**b**), ML outputs (**c**–**e**), and plots of the Euclidian differences (**f**,**g**) obtained from the comparison between 2012 and 2018 DTMs of the study area. Dam construction area is shown here.

**Figure 15:**
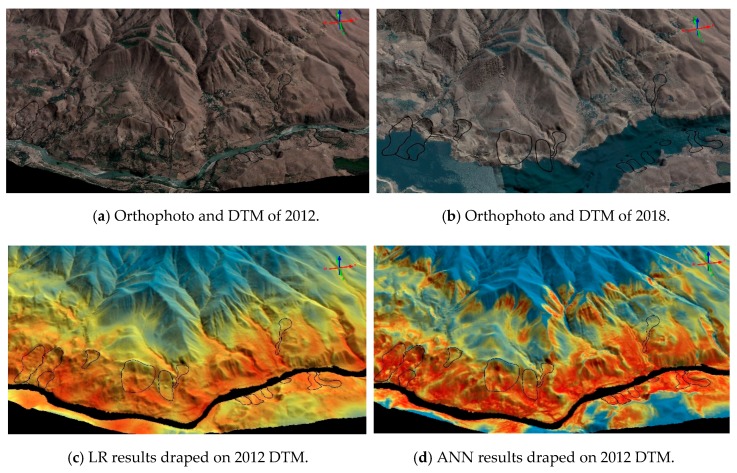

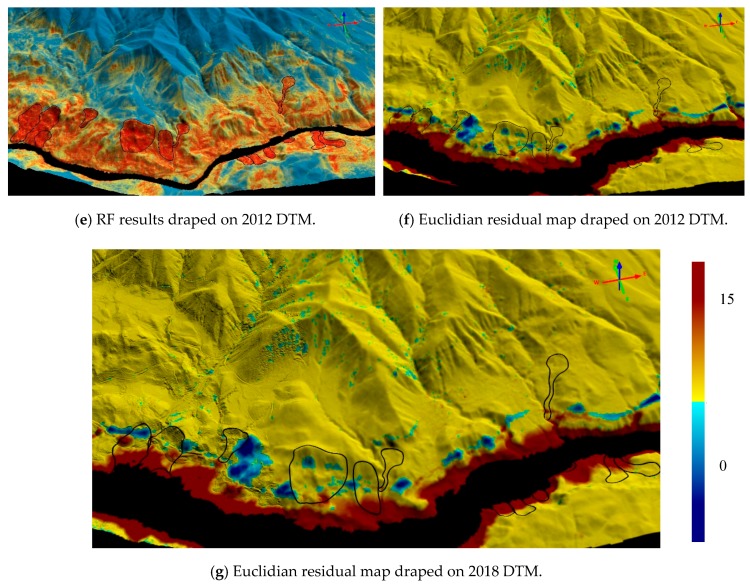
The ortophotos (**a**,**b**), ML outputs (**c**–**e**), and plots of the Euclidian differences (**f**,**g**) obtained from the comparison between 2012 and 2018 DTMs of the study area. The road area is shown here.

The surface comparison, as the novel part of the study, was performed between the 2012 and 2018 DTMs. The latter one was produced from the images acquired after the dam reservoir has been filled. Therefore, both construction efforts and landslides cause differences between both terrain models. The Euclidian distance residuals plot of the whole area obtained from LS3D are provided in Figure 13. The red and the blue areas in Figure 13 demonstrate the highest elevation changes. Two parts of the study area, one that comprise the road and the other one that comprise the dam construction site were analyzed visually and screenshots showing the performances of each ML and the terrain deformations are shown in Figure 14 and Figure 15. The darker blue areas in all residual plots represent landslides, although part of those are affected by the road construction. All ML methods used here detect the landslide areas, although LR and ANN methods are more conservative than the RF method as can be seen from Figure 14 and Figure 15.

## 5. Discussions and Conclusions

Regional landslide susceptibility assessment is one of the hot topics in the international literature since these assessments are used for landslide hazard mitigation efforts. Depending on advancements of the ML techniques, the performances of these assessments have improved significantly. However, during the performance assessment stage, existing landslide inventories have been used as an obligation. Similarly, for the model performance, the landslide inventory before 2012 was used. However, in the present study, landslide susceptibility maps were produced using the DTM of 2012 (before construction of the dam), and the DTM of 2018 (after construction of the dam) was used for comparison. Such comparison provides more realistic assessment because the main purpose of a landslide susceptibility map is to predict future landslides. In the present study, fully completed datasets were used for this purpose. The results and conclusions obtained from the present study can be drawn as follows:

1. Considering the geological and geomorphological characteristics of the study area, it is evident that the reservoir area is highly susceptible to landsliding, and a total of 46 landslides were mapped employing orthophotos of 2012. The landslide inventory was checked with extensive field observations. The study area is generally covered by sparse vegetation cover, and the landslides in the study area can be easily observed. Consequently, the reliability of the landslide inventory is high. The performance of a data-driven method is directly based on the quality of the data and the suitability of the method used.
2. Three landslide susceptibility maps were produced by applying three different ML techniques (LS, ANN, and RF). Considering the ROC assessment, it is possible to say that all maps show good performance. However, the RF method yields the best classification accuracy with an area value of 0.95, where ANN comes second in the performance with an area of 0.84.
3. Based on the surface comparison analysis, the most conservative results were given by LR, while the most representative results were provided by RF. Consequently, the RF is the most appropriate approach for the area considered in the present study. These results are supported by the model performance assessment. In other words, there is a good agreement between AUC values and Euclidean distance calculations introduced in the present study.
4. Performance assessment of landslide susceptibility map is still one of major research areas among the international landslide community. For this reason, such type cases are extremely important for accurate assessment of performances of the existing techniques. In other words, if landslide susceptibility maps are produced before occurring a triggering event, performance assessment can be performed accurately after triggering event using Euclidean distance calculations.
5. In addition to developing technologies and methods, the presence of more and representative historical data makes landslide susceptibility maps more efficient. Therefore, landslide susceptibility maps are becoming more effective and useful tools for reducing losses caused by landslides. Some recent studies [57,58] described new approaches (CitSci) and new techniques (Convolutional Neural Networks) to collect the landslide data and to check the quality of the data. Consequently, the approach introduced in the present study has promising results for increasing the quality of landslide susceptibility maps because of the increase in the amount and quality of data.

## Figures and Tables

**Table 1:** Descriptive statistics of the landslide area, volume, and expected depth of failures.

Statistics	Area (m^2^)	Volume (m^3^)	Expected Depth of Failure (m)
Min.	9725.78	29,941.13	18.47
Max.	382,327.00	3,982,504.13	62.50
Mean	71,114.29	506,904.93	32.68
Std. Deviation	77,731.99	802,017.70	10.07

**Table 2:** Main specifications of the photogrammetric flight missions from 2012 and 2018.

Flight Year	Number of Photos	Flight Altitudes	GSD	Forward Lap & Side Lap	Camera	Image Orientation Accuracy
2012	34	8300 m–8900 m	54 cm–58 cm	~60% ~20%	Ultracam Eagle from Vexcel Imaging	Planimetry: 1/3 GSD Height: 1/2 GSD
2018	52	6650 m–6750 m	43 cm–50 cm	~70% ~30%		

**Table 3:** Statistical summary of the values calculated from the topographic derivative parameters.

Parameter	Max	Min	Mean	Median	Standard Deviation	Skewness	Kurtosis
Aspect	6.283	0.000	3.125	3.162	1.752	−0.017	−1.090
Distance to Channel	678.8	0.000	113.923	94.868	87.933	1.092	1.513
Distance to Ridge	492.5	0.000	102.212	89.443	76.0	0.893	0.529
Elevations	2060.8	1011.5	1418.4	1373.6	211.7	1.043	0.704
General Curvature	2.249	−2.943	0.000	0.003	0.079	−1.551	35.520
Plan Curvature	0.079	−0.090	0.000	0.000	0.009	−0.154	2.039
Profile Curvature	0.105	−0.099	0.000	0.000	0.008	−0.505	3.921
Slope	1.329	0.000	0.361	0.362	0.192	0.155	−0.701
SPI	7,899,360.0	0.000	717.213	29.776	22,157.900	166.126	38,897.104
TWI	24.061	0.794	5.297	4.855	1.991	2.523	11.358

**Table 4:** The model parameters used for ANN, logistic regression (LR), and random forest (RF) methods.

Model	Parameter	Value
Logistic Regression	Max iteration	1000
	Loss function	Binary Cross Entropy with L2
Artifical Neural Network	Max iteration	1000
	Learning Rate	0.01
	Optimization algorithm	ADAM
	Activation Function	Sigmoid
	Loss Function	Binary Cross Entropy with L2
Random Forest	Number of trees	500
